# Impact of access to coronary angiography and percutaneous coronary intervention on in-hospital and five-year mortality in patients with acute coronary syndrome: a propensity-matched cohort study in Thailand

**DOI:** 10.1186/s41256-024-00390-x

**Published:** 2024-11-19

**Authors:** Ponlagrit Kumwichar, Jutatip Thungthong, Tippawan Liabsuetrakul, Hisateru Tachimori, Mariko Hosozawa, Eiko Saito, Yuta Taniguchi, Virasakdi Chongsuvivatwong, Hiroyasu Iso

**Affiliations:** 1 Department of Epidemiology, Faculty of Medicine, Prince of Songkla University, Hat Yai, Songkhla Thailand; 2 National Health Security Office, Nonthaburi, Thailand; 3 National Center for Global Health and Medicine, Institute for Global Health Policy Research, Tokyo, Japan; 4 Endowed Course for Health System Innovation, Keio University School of Medicine, Tokyo, Japan

**Keywords:** Healthcare services, Quality of care, Access to care, National database utilization

## Abstract

**Background:**

Coronary artery angiography (CAG) and percutaneous coronary intervention (PCI) are superior to non-invasive approaches in reducing mortality in patients with ST-segment elevation myocardial infarction (STEMI). However, their efficacy remains uncertain in non-ST-elevation acute coronary syndromes (NSTE-ACS) and limited in low-resource settings. This study aimed to compare in-hospital and 5-year mortality rates between patients with a first event of STEMI and NSTE-ACS who underwent CAG and PCI and those with similar severity who did not undergo CAG and PCI.

**Methods:**

A propensity-matched retrospective cohort study was conducted using population-based claims data of national universal coverage of Thailand for identification of patients with acute coronary syndromes. The mortality of recruited patients was additionally linked to the national database of vital registration. Patients aged ≥ 40 years who were hospitalized for STEMI and NSTE-ACS in 2017, with a focus on access to CAG and PCI were included. For each condition either STEMI or NSTE-ACS, patients who underwent CAG and PCI were matched to those who did not undergo using propensity score matching (PSM) to balance measured confounders, such as age, sex, and underlying conditions. In-hospital mortality rate ratio and 5-year mortality were analyzed as measures.

**Results:**

Through PSM, 2,702 non-intervention STEMI patients were paired with an equal number of intervention patients, and similarly, 5,072 non-intervention NSTE-ACS patients were matched with an equivalent group who received interventions. For patients with STEMI, the in-hospital mortality rate ratio (95% confidence interval (CI)) for those who underwent CAG and PCI compared to those who did not was 30.1% (30.0%, 30.2%). Similar trends were observed in patients with NSTE-ACS with a mortality rate of 34.7% (34.6%, 34.8%). For the five-year mortality comparison, the hazard ratios (95% CI) of mortality after discharge were 0.55 (0.50, 0.62) for STEMI and 0.57 (0.54, 0.61) for NSTE-ACS cases.

**Conclusions:**

Access to CAG and PCI was significantly associated with lower in-hospital and 5-year mortality rates in patients who experienced their first event of ACS, despite the limited availability of some unmeasured or residual confounders. Healthcare systems should expand their resources for CAG and PCI in Thailand and other countries to equitably enhance longevity.

**Supplementary Information:**

The online version contains supplementary material available at 10.1186/s41256-024-00390-x.

## Introduction

Acute coronary syndrome (ACS) is a prevalent cardiovascular disease and a leading global cause of death [1]. Approximately 75% of ACS-related deaths occur in low- and middle-income countries [2], which is related to aging populations, sedentary lifestyles, unhealthy diets, and limited resources [3]. In Thailand, ACS has consistently ranked as the leading cause of death and disability-adjusted life years over the past decade [4, 5]. Coronary artery angiography (CAG) and percutaneous coronary intervention (PCI) are the preferred procedures for the diagnosis and treatment of patients diagnosed with ST-segment elevation myocardial infarction (STEMI) [6] and have been proven more effective than thrombolytic therapy [7]. Patients with non-ST-elevation ACS (NSTE-ACS) with high-risk features also require coronary angiography and PCI [6]. Research from the United States, United Kingdom, and Sweden has shown that the primary utilization of PCI has been increasing among patients with STEMI since 2004 [8, 9]. This trend is also observed in Thailand, where PCI usage for patients with STEMI rose from 21.9% in 2011 to 53.9% in 2017 [10]. Although 18-month follow-up outcomes are well-studied [7, 11, 12], research on long-term survival after PCI over two years is still limited. Most PCI-effectiveness studies have been conducted in Western countries [8, 9, 11] in which healthcare structures and socioeconomic status differ from those in Thailand [10].

Unlike wealthy nations, Thailand, which has a low-resource setting, faces challenges in providing equitable support for CAG and PCI for all patients with ACS. From 2011 to 2017, the National Health Security Office (NHSO) in Thailand identified preliminary evidence suggesting that enhancing access to PCI for patients with STEMI could notably reduce mortality rates over time [10]. Since 2017, the NHSO has endeavored to facilitate reimbursement for all patients requiring CAG and PCI, including those with STEMI and NSTE-ACS. Despite these efforts, a discrepancy exists between the number of cases and the capacity of the health system. As a result, some patients who need CAG and PCI have been unable to access such services. The effect of disparities in access to CAG and PCI on patient survival remains unclear. This knowledge gap underscores the necessity for real-world data analyses to elucidate the decreased mortality rate associated with access to CAG and PCI.

Thailand is an upper-middle-income country with three primary healthcare schemes: civil servant medical benefits, social security, and universal coverage (UC). For ACS, the claims data of UC (accounting for over 75% of cases) are collected by the NHSO [13] and the health facilities, have been reimbursed for the costs of thrombolytic agents and instruments for CAG and PCI, since 2009 using fee schedules [10]. Additionally, the claims databases of the NHSO are linked to the national database of vital registration (VR) [14], thereby enabling the tracking of mortality. In this study, we aimed to compare the in-hospital and five-year mortality rates of patients with a first event of STEMI and NSTE-ACS who could access CAG and PCI with those who could not. By understanding the benefits of access to CAG and PCI in prolonging lifespan on a national scale, we can advocate for policymakers in the health system to enhance access to CAG and PCI for all patients experiencing their first ACS event. This approach ensures equity and upholds human rights to promote longevity [15].

## Methods

### Study design

This study utilized a retrospective cohort design, selecting individuals hospitalized due to STEMI or NSTE-ACS from claims databases in 2017 and analyzing each condition separately. We focused on access to CAG and PCI, using no access to these as the reference. All individuals were tracked in the VR database until September 30, 2023, using the relevant databases.

### Data sources and enrolled participants

Inpatient claims and VR databases were linked using encrypted identification numbers. Using the International Classification of Diseases, Tenth Revision (ICD-10), we retrieved only the data of patients with a primary diagnosis of STEMI (I21.0-3) and NSTE-ACS (I21.4) who were hospitalized in PCI-center hospitals. We selected these ICD-10 codes to eliminate confounding from repeat events and to ensure a more focused analysis on the first presentation of ACS in our study. To obtain the most updated situation of CAG and PCI services with a 5-year follow-up, we selected only patients hospitalized due to a first event of STEMI or NSTE-ACS between January 1 and December 31, 2017.

Considering the rarity of ACS in patients aged < 40 years, [16] such patients were excluded from our analysis. As indicated by the ICD, Ninth Revision, Clinical Modification (ICD-9-CM) codes, our study excluded individuals who had previously undergone PCI (00.40–00.48, 00.66, and 36.06–36.07) or coronary artery bypass grafting (CABG) procedures (36.10–36.19). Additionally, individuals with a history of hospitalization due to any form of ischemic heart disease, classified under diagnoses I20-25, prior to January 1, 2017 (since 2011) were excluded. The exclusion criteria were also extended to those who received CABG as the initial definitive treatment for their first ACS event.

### Exposure and confounders

Using ICD-10 codes, individuals were stratified into two groups: STEMI identified by I21.0-3 and NSTE-ACS identified by I21.4. Within each stratum, access to CAG and PCI was the exposure of interest, as determined by the ICD-9-CM codes for CAG (88.5-7) and PCI (00.40–00.48, 00.66, and 36.06–36.07). Individuals who received medical treatment without undergoing coronary procedures were included in the control group. Within the STEMI stratum, nearly all patients in the unexposed group (99%) underwent revascularization with streptokinase, whereas only 1% were treated with a recombinant tissue plasminogen activator agent.

Age and sex were obtained from the VR database as confounding variables. The underlying diseases were identified through data mining for any ICD-10 code in the claims database prior to the date of hospitalization for the first ACS event, as shown in sTable 1 (Supplementary Table 1). In the STEMI group, the purpose of admission was classified as waiting for the evaluation of primary and rescue PCI. In the NSTE-ACS group, the reasons for admission included direct admission from any department of the PCI-center hospital and referral from another hospital for CAG consideration. These referral cases were severe, necessitating urgent transfer to a PCI-center hospital for further evaluation. As clinical assessment data were not available in the claims databases, this variable was used as a surrogate for assessing the high-risk group for NSTE-ACS. The health zonal region in Thailand is also a potential confounder, as people in each region have different lifestyles associated with mortality [17] and there is a varying capacity for handling cases of CAG and PCI [10]. We identified the health zonal region based on the PCI-center hospital where the patients were hospitalized.

### Outcomes

The outcomes of interest were in-hospital death and death after discharge; however, if our analysis only considered any death after discharge as an outcome, the results might be biased due to deaths from causes unrelated to heart conditions, a phenomenon known as collider bias [18]. Therefore, we analyzed both deaths from any cause and deaths related to heart conditions, as documented in the VR database. Causes of death are recorded in Thai as reported in clinical autopsies and verbal autopsies when clinical autopsies are not feasible. Deaths related to heart conditions were identified through the cause of death recorded in the VR database in Thai, specifically any text containing “หัวใจ',” which translates to “heart.”

### Propensity score matching

For patients with STEMI and NSTE-ACS, our goal was to balance the mortality risk between those who underwent CAG and PCI and those who did not. We performed 1:1 propensity score matching (PSM) using an optimal matching algorithm [19] for age, sex, underlying diseases, purpose of admission, and health zone regions. Individuals who did not meet the inclusion criteria were excluded from the study. Theoretically, we hypothesized that groups with and without access to CAG and PCI would exhibit comparable characteristics and baseline survival risks after matching.

### Statistical analysis

For both pre- and post-matched groups, demographic information was systematically summarized using descriptive statistical methods to compare the baseline characteristics of patients with and without access to CAG and PCI. This comparison was facilitated by applying a standardized mean difference (SMD) metric. An SMD value exceeding 0.1 was deemed to be statistically significant [20]. After balancing all measured confounders through PSM, we designated patients without access to CAG and PCI as the reference group. We then compared this reference group with the exposed group in terms of three outcomes: in-hospital death, death from any cause after discharge, and death related to heart conditions after discharge. To compare in-hospital mortality rates, we computed mortality rate ratios, subsequently utilizing the logarithmic transformation method to derive the 95% confidence interval (95% CI) [21].

Post-discharge mortality was analyzed using a time-to-event approach. Patients who experienced in-hospital death were excluded. For this analysis, all participants were censored at the completion of the 1,825-day follow-up period or on the date of death. Thus, the number of patients at risk was consistent over time for both outcomes despite differing event numbers. A Kaplan–Meier plot was used to visualize survival trends over time for both outcomes after discharge. Cox regression analysis was used to compare the hazard ratios (HRs) between the exposed and reference groups. The parallelism of a log-minus-log plot of the hazard function was used to verify the proportional hazards assumption [22]. Since the observed association in our analysis could be attributed to unmeasured or residual confounders, we calculated the “E-value.” This represents the minimum strength of association required between access to coronary procedures (CAG + PCI) and mortality for an unmeasured or residual confounder to explain the observed association. A notably high E-value suggests that it is implausible for an unmeasured or residual confounder to explain the findings [23].

All analyses were performed using the MatchIt [24], epiDisplay [25], survminer [26], tidyverse [27], and EValue [28] packages in the R language and environment (version 4.1.1; R Core Team, Vienna, Austria). Statistical significance was set at *p* < 0.05.

## Results

### Characterisitcs of patients recruited

The selection and stratification processes for the cohort study of patients hospitalized with ischemic heart disease between January 1 and December 31, 2017, are presented in sFigure 1 (Supplementary Fig. 1). Initially, 39,944 patients were included, and after exclusion, 34,606 remaining patients were stratified into two groups based on their primary diagnosis at admission: STEMI and NSTE-ACS. Of the 10,925 patients with STEMI, 930 who underwent CAG without PCI were excluded. Using PSM, 2,702 non-intervention patients were matched with an equal number of patients who underwent interventions. Similarly, of the 23,681 patients with NSTE-ACS, 2,868 who underwent CAG without PCI were excluded. Another PSM process matched 5,072 non-intervention patients with an equal number of intervention patients.

A comparison of the characteristics of patients experiencing their first STEMI event before and after PSM is presented in Table 1. Initially, 7,293 patients with access to CAG and PCI were compared with 2,702 patients across various demographics. After matching, 2,702 patients from each group were compared, showing closer alignment of characteristics, as indicated by SMD values less than 0.1. Table 2 compares the characteristics of patients who experienced their first NSTE-ACS event before and after PSM. Initially, it compared 5,072 patients with access to CAG and PCI with 15,741 patients without access, considering various demographics and health conditions. After matching, each group consisted of 5,072 patients, revealing a closer alignment of characteristics.

**Table 1:** Comparison of characteristics of patients with first STEMI event pre- and post-matching for access to CAG and PCI

Demographics	Pre-matched			Post-matched		
	Access to CAG and PCI	No access to CAG and PCI	SMD	Access to CAG and PCI	No access to CAG and PCI	SMD
Total, N	7293	2702		2702	2702	
n (%)						
Male	5209 (71.4)	1756 (65.0)	0.139	1799 (66.6)	1756 (65.0)	0.034
Age, mean ± SD	63.6 ± 11.4	66.5 ± 12.9	0.236	65.8 ± 12.0	66.5 ± 12.9	0.052
Hypertension	3501 (48.0)	1152 (42.6)	0.108	1128 (41.7)	1152 (42.6)	0.018
Hypercholesterolemia	3342 (45.8)	927 (34.3)	0.237	935 (34.6)	927 (34.3)	0.006
Diabetes	2064 (28.3)	662 (24.5)	0.086	671 (24.8)	662 (24.5)	0.008
Hyperthyroidism	38 (0.5)	25 (0.9)	0.048	25 (0.9)	25 (0.9)	< 0.001
Hypothyroidism	81 (1.1)	38 (1.4)	0.027	36 (1.3)	38 (1.4)	0.006
Chronic kidney disease	626 (8.6)	365 (13.5)	0.158	332 (12.3)	365 (13.5)	0.036
Severe obesity	28 (0.4)	6 (0.2)	0.029	5 (0.2)	6 (0.2)	0.008
Valvular heart disease	228 (3.1)	84 (3.1)	0.001	85 (3.1)	84 (3.1)	0.002
Previous TIA/stroke	221 (3.0)	160 (5.9)	0.14	132 (4.9)	160 (5.9)	0.046
Atrial fibrillation	491 (6.7)	165 (6.1)	0.026	184 (6.8)	165 (6.1)	0.029
Heart failure	1305 (17.9)	620 (22.9)	0.126	605 (22.4)	620 (22.9)	0.013
Purpose of admission at a PCI-center hospital			*0.348*			*0.045*
To consider primary PCI	6142 (84.2)	142 (5.3)		116 (4.3)	142 (5.3)	
To consider rescue PCI	1151 (15.8)	2560 (94.7)		2586 (95.7)	2560 (94.7)	
Health regional zone			*0.632*			*0.078*
1	687 (9.4)	286 (10.6)		318 (11.8)	286 (10.6)	
2	400 (5.5)	139 (5.1)		151 (5.6)	139 (5.1)	
3	352 (4.8)	118 (4.4)		120 (4.4)	118 (4.4)	
4	642 (8.8)	123 (4.6)		115 (4.3)	123 (4.6)	
5	607 (8.3)	240 (8.9)		252 (9.3)	240 (8.9)	
6	651 (8.9)	164 (6.1)		167 (6.2)	164 (6.1)	
7	578 (7.9)	104 (3.8)		113 (4.2)	104 (3.8)	
8	524 (7.2)	124 (4.6)		116 (4.3)	124 (4.6)	
9	725 (9.9)	126 (4.7)		127 (4.7)	126 (4.7)	
10	410 (5.6)	153 (5.7)		171 (6.3)	153 (5.7)	
11	450 (6.2)	459 (17)		403 (14.9)	459 (17.0)	
12	596 (8.2)	554 (20.5)		541 (20.0)	554 (20.5)	
13	671 (9.2)	112 (4.1)		108 (4.0)	112 (4.1)	

CAG, coronary artery angiography; PCI, percutaneous coronary intervention; SD, standard deviation; STEMI, ST-segment elevation myocardial infarction; SMD, standardized mean difference; TIA, transient ischemic attack

**Table 2:** Comparison of characteristics of patients with first NSTE-ACS event pre- and post-matching for access to CAG and PCI

**Demographics**	**Pre-matched**			**Post-matched**		
	Access to CAG and PCI	No access to CAG and PCI	SMD	Access to CAG and PCI	No access to CAG and PCI	SMD
Total, N	5072	15,741		5072	5072	
n (%)						
Male	3061 (60.4)	8057 (51.2)	0.185	3061 (60.4)	3025 (59.6)	0.014
Age, mean ± SD	65.0 ± 10.8	69.0 ± 12.2	0.35	65.0 ± 10.8	65.1 ± 12.1	0.009
Hypertension	3639 (71.7)	9016 (57.3)	0.306	3639 (71.7)	3576 (70.5)	0.027
Hypercholesterolemia	3300 (65.1)	6018 (38.2)	0.557	3300 (65.1)	3242 (63.9)	0.024
Diabetes	2131 (42.0)	5599 (35.6)	0.133	2131 (42.0)	2158 (42.5)	0.011
Hyperthyroidism	54 (1.1)	205 (1.3)	0.022	54 (1.1)	54 (1.1)	< 0.001
Hypothyroidism	109 (2.1)	388 (2.5)	0.021	109 (2.1)	113 (2.2)	0.005
Chronic kidney disease	951 (18.8)	4213 (26.8)	0.192	951 (18.8)	989 (19.5)	0.019
Severe obesity	35 (0.7)	51 (0.3)	0.052	35 (0.7)	26 (0.5)	0.023
Valvular heart disease	285 (5.6)	967 (6.1)	0.022	285 (5.6)	263 (5.2)	0.019
Previous TIA/stroke	179 (3.5)	605 (3.8)	0.017	179 (3.5)	190 (3.7)	0.012
Atrial fibrillation	403 (7.9)	1730 (11.0)	0.104	403 (7.9)	414 (8.2)	0.008
Heart failure	1473 (29.0)	6064 (38.5)	0.201	1473 (29.0)	1476 (29.1)	0.001
Type of admission at a PCI-center hospital			*0.236*			*0.071*
From any department	4598 (90.7)	15,176 (96.4)		4598 (90.7)	4698 (92.6)	
Referred from other hospital	474 (9.3)	565 (3.6)		474 (9.3)	374 (7.4)	
Health regional zone			*0.437*			*0.089*
1	381 (7.5)	1302 (8.3)		381 (7.5)	399 (7.9)	
2	279 (5.5)	790 (5.0)		279 (5.5)	295 (5.8)	
3	265 (5.2)	747 (4.7)		265 (5.2)	270 (5.3)	
4	566 (11.2)	1324 (8.4)		566 (11.2)	585 (11.5)	
5	579 (11.4)	1432 (9.1)		579 (11.4)	591 (11.7)	
6	487 (9.6)	1363 (8.7)		487 (9.6)	498 (9.8)	
7	324 (6.4)	891 (5.7)		324 (6.4)	324 (6.4)	
8	255 (5.0)	1250 (7.9)		255 (5.0)	293 (5.8)	
9	399 (7.9)	1500 (9.5)		399 (7.9)	389 (7.7)	
10	212 (4.2)	1040 (6.6)		212 (4.2)	239 (4.7)	
11	318 (6.3)	1647 (10.5)		318 (6.3)	322 (6.3)	
12	273 (5.4)	1627 (10.3)		273 (5.4)	271 (5.3)	
13	734 (14.5)	828 (5.3)		734 (14.5)	596 (11.8)	

CAG, coronary artery angiography; NSTE-ACS, non-ST-elevation acute coronary syndrome; PCI, percutaneous coronary intervention; SD, standard deviation; SMD, standardized mean difference; TIA, transient ischemic attack

### In-hospital mortality

Table 3 presents mortality rate ratios for patients with two types of ACS based on their access to CAG and PCI. In the case of STEMI, patients with access to CAG and PCI had a mortality rate of 102.1 deaths per 1,000 patients, significantly lower than the 339.4 deaths per 1,000 patients in the group without access, resulting in a mortality rate ratio (95% CI) of 30.1% (30.0%, 30.2%). For NSTE-ACS, patients with access had a mortality rate of 32.9 deaths per 1,000 patients, compared to 94.8 deaths per 1,000 patients in the no access group, with a mortality rate ratio (95% CI) of 34.7% (34.6%, 34.8%).

**Table 3:** In-hospital mortality rate ratios of the patients hospitalized for STEMI and NSTE-ACS

Type of ACS	Access to CAG and PCI		No access to CAG and PCI (ref.)		Mortality rate ratios (%) (95% CI)
	In-hospital death	Deaths per 1000 patients	In-hospital death	Deaths per 1000 patients	
STEMI	276/2702	102.1	917/2702	339.4	30.1 (30.0, 30.2)
NSTE-ACS	167/5072	32.9	481/5072	94.8	34.7 (34.6, 34.8)

ACS, acute coronary syndrome; CAG, coronary artery angiography; CI, confidence interval; NSTE-ACS, non-ST-elevation acute coronary syndrome; PCI, percutaneous coronary intervention; STEMI, ST-segment elevation myocardial infarction

### Five-year mortality after discharge

After excluding those who died in-hospital, among the patients with STEMI, the remaining total was 4,211, with 1,785 (42.4%) not undergoing CAG and PCI and 2,426 (57.6%) undergoing CAG and PCI. For patients with NSTE-ACS, the remaining number was 9,496, with 4,591 (48.3%) not undergoing CAG and PCI and 4,905 (51.7%) undergoing CAG and PCI. We re-evaluated the balance of characteristics after post-exclusion and identified an imbalance solely in the distribution of healthy zonal areas, caused specifically by an imbalance in the 11th zonal area. This led to an SMD greater than 0.1 but less than 0.3, as detailed in sTable 2 and sTable 3 (Supplementary Tables 2–3), which is considered acceptable. Hence, we continued the time-to-event analysis without any rematching.

**Fig. 1:**
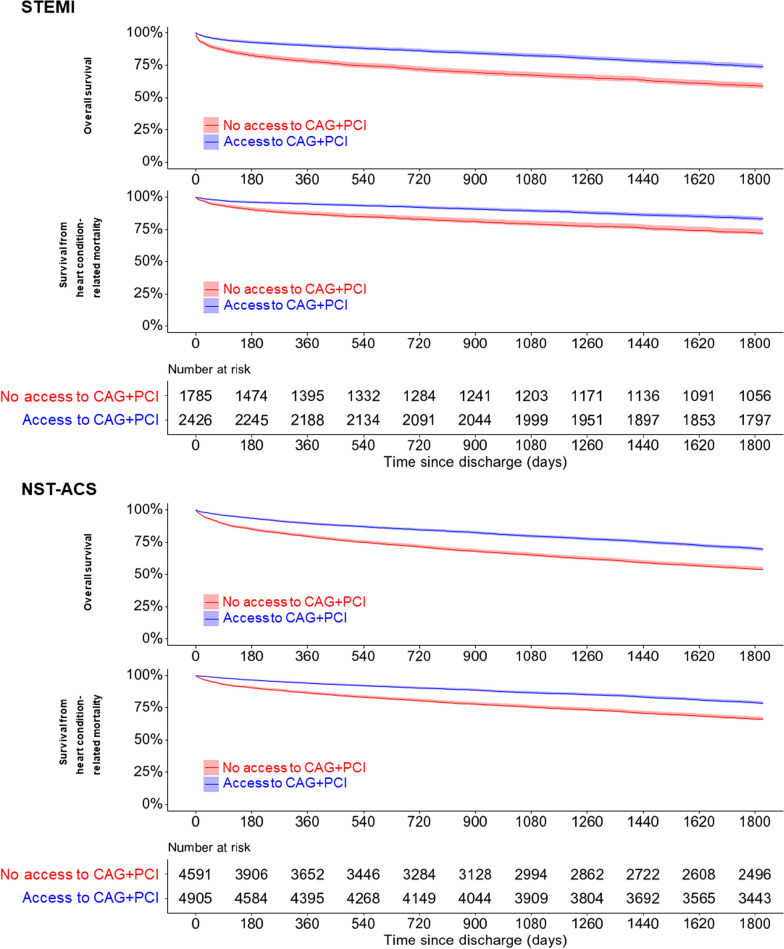
Comparison of overall survival and heart condition-related mortality between patients with and without access to CAG and PCI, for STEMI and NSTE-ACS cases. *Legends:* CAG, coronary artery angiography; PCI, percutaneous coronary intervention; STEMI, ST-segment elevation myocardial infarction; ACS, acute coronary syndrome

Figure 1 presents the Kaplan–Meier survival curves, including 95% CI bars, comparing overall survival and heart condition-related mortality for STEMI and NSTE-ACS cases between patients with access to CAG and PCI (blue) and those without (red). The survival curves suggest that patients with access to CAG and PCI exhibited a higher survival rate over the observed period of 1,825 days, with both overall survival and specific survival from heart condition-related mortality outperforming that of those without access.

The HRs for the overall and heart condition-related mortality in patients with STEMI and NSTE-ACS are shown in Table 4. Access to CAG and PCI was significantly associated with a 45% lower risk of mortality from STEMI (HR = 0.55) and a 43% reduction in NSTE-ACS (HR = 0.57) than those without access. The parallelism of a log-minus-log plot of the hazard function for all models, indicating no violation of the proportional hazard assumption as shown in sFigure 2 to sFigure 5 (Supplementary Figs. 2–5).

**Table 4:** Hazard ratios for overall and heart condition-related mortality in patients with ACS with and without access to CAG and PCI

Outcome	Type of ACS	Events per person-years		HR (95% CI)
		Access to CAG and PCI	No access to CAG and PCI (ref.)	
Overall mortality	STEMI	638/10210	737/6314	0.55 (0.50, 0.62)
	NSTE-ACS	1490/20217	2104/15945	0.57 (0.54, 0.61)
Heart condition-related mortality	STEMI	380/10210	440/6314	0.55 (0.48, 0.63)
	NSTE-ACS	985/20217	1389/15945	0.57 (0.53, 0.62)

ACS, acute coronary syndrome; CAG, coronary artery angiography; CI, confidence interval; HR, hazard ratio; NSTE-ACS, non-ST-elevation acute coronary syndrome; PCI, percutaneous coronary intervention; STEMI, ST-segment elevation myocardial infarction

### Sensitivity analysis

In-hospital mortality was observed to have an E-value of 6.1 among patients admitted for STEMI and 5.2 for those admitted for NSTE-ACS. Regarding five-year overall or condition-specific mortality, the E-values for the HR were 2.4 for STEMI admissions and 2.3 for NSTE-ACS. Our literature review suggests that the five-year mortality rates might be attributable to unmeasured confounders of current smoking status. This factor exhibits a relative risk of 3.06 for cardiac death [29], which surpasses the E-value for five-year mortality rates. Thus, it is plausible that current smokers are less likely to undergo CAG and PCI, contributing to increased 5-year mortality rates. However, our review did not identify any confounders that could account for the decreased in-hospital mortality associated with access to CAG and PCI.

## Discussion

In this study, after balancing potential confounders, patients with access to CAG and PCI experienced significantly lower in-hospital mortality rates in both the STEMI and NSTE-ACS groups than those without access, as demonstrated by the mortality rate ratios. A 5-year follow-up indicated that access to CAG and PCI was associated with improved overall survival and reduced heart condition-related mortality. The consistency of these results suggests that external causes of death are less likely to affect long-term mortality. HRs further confirmed the substantial reduction in mortality risk among patients with access to CAG and PCI, underscoring the importance of access to CAG and PCI in prolonging longevity. Access to CAG and PCI could reduce mortality by approximately 70% during hospitalization and by 50% over five years. Our sensitivity analysis suggested a robust association between access to CAG and PCI and decreased in-hospital mortality in patients with ACS. However, the association between access to CAG and PCI and decreased five-year mortality in patients with ACS remains inconclusive because it could be explained by smoking, as not measured in our study, which potentially confounds the results by indication [30, 31]. It is widely recognized that understanding the reasons behind treatment decisions within existing claim data is challenging, particularly with clinical complexity like ACS in relation to CAG and PCI.

The reduction in in-hospital mortality rates among patients with STEMI accessing CAG and PCI is partially attributed to the comparison with the reference group treated with streptokinase, which has less than a 50% chance of reperfusion within 90 min [32]. In contrast, the definitive lesion-identification capabilities of CAG and the subsequent targeted treatment offered by PCI underscore its superiority over the poorly effective fibrinolytic therapy provided by streptokinase [32, 33]. Additionally, knowledge obtained from CAG regarding the lesion can guide cardiologists to manage STEMI and NSTE-ACS cases more appropriately [34].

Although the association between access to CAG and PCI and a reduction in five-year mortality rates was not robust, our findings corroborate those of other studies on STEMI in high-income countries [35, 36]. These studies have uniformly illustrated the superiority of CAG and PCI over pharmacotherapy alone for the management of ACS. Access to CAG and PCI at the first ACS event plausibly enhance care quality, directly influencing the myocardial infarction area. Minimizing the infarction size from the beginning is likely to decrease long-term mortality rates. However, a meta-analysis of randomized controlled trials indicated that there was no decrease in the long-term mortality rates for NSTE-ACS after CAG and PCI [37]. This difference may stem from the fact that unmeasured confounders, including current smoking, were not balanced in our study. A study using a database from Korean National Health Insurance System collected the information of smoking status before and after PCI which showed the influence of smoking on all-cause death [31]. Therefore, we recommend that mandatory smoking data be included in the NHSO records.

The systems for CAG and PCI in STEMI and NSTE-ACS patients under Thailand’s universal coverage scheme differ from those in other countries, limiting direct comparisons. There were some limitations of this study. First, the data on the time to revascularization intervention and severity assessment were not presented as the variables were not recorded in the database. Hospital information was encrypted, making it impossible to identify hospital levels. However, the sensitivity analysis suggests that these unmeasured confounders are unlikely to explain the significant findings observed. Second, this study did not include patients with ACS who died before hospitalization and focused only on patients experiencing their first ACS event. This may have led to an underestimation of both the number of patients who underwent CAG and PCI and those who did not, though this likely represents only a small minority. Third, the use of existing ICD-10 coding for diagnosis may introduce some errors; however, these are expected to be minimal since all medical claims and laboratory data at the hospital level are reviewed and audited before being finalized and recorded in the NHSO database. Fourth, when examining five-year mortality, the results may be influenced by current smoking status, which we were unable to track. This limitation should be addressed in future research. Fifth, we could not obtain all hospital visit data, which meant that we could not perform more detailed analyses considering the events after discharge. Finally, the effects of COVID-19 infection on 5-year mortality in these groups of patients from 2020 onwards could not be measured due to the lack of additional data. However, the survival hazards were not strikingly different between the periods before and after 2020.

## Conclusions

In low-resource settings, access to CAG and PCI was significantly associated with reduced mortality risk in patients experiencing their first ACS event. This association was observed both during hospitalization and over a 5-year follow-up period, despite the potential limitations of unmeasured or residual confounders. Therefore, it is imperative for healthcare systems to enhance public education, ambulance infrastructure, and human resources to improve access to CAG and PCI and reduce inequities in care. Further research is needed on the indications for these procedures and on clinical monitoring systems, including cost-effectiveness analyses, to establish an appropriate model for policy implementation.

## Supplementary Information

Additional file 1.

## Data Availability

The data for the dynamic cohort and R codes used in the analysis are available in the GitHub repository. Only users with permission from the NHSO can access this dataset. If you require access, please contact the NHSO of Thailand for permission. For the GitHub repository used in this study, please refer to https://github.com/ponlagrit/PCI_access.

## Abbreviations

ACS: Acute coronary syndrome
CI: Confidence interval
CAG: Coronary artery angiography
ICD-10: International Classification of Diseases, Tenth Revision
ICD-9-CM: ICD, Ninth Revision, Clinical Modification
NSTE-ACS: Non-ST-elevation acute coronary syndromes
PCI: Percutaneous coronary intervention
PSM: Propensity score matching
SMD: Standardized mean difference
STEMI: ST-segment elevation myocardial infarction
NHSO: National Health Security Office
UC: Universal coverage
VR: Vital registration

## References

[1] GBD 2017 Causes of Death Collaborators. Global, regional, and national age-sex-specific mortality for 282 causes of death in 195 countries and territories, 1980–2017: a systematic analysis for the Global Burden of Disease Study 2017. Lancet. 2018;392(10159):1736–88. 30496103 10.1016/S0140-6736(18)32203-7 PMC6227606

[2] Roth GA, Johnson C, Abajobir A, Abd-Allah F, Abera SF, Abyu G, et al. Global, regional, and national burden of cardiovascular diseases for 10 causes, 1990 to 2015. J Am Coll Cardiol. 2017;70(1):1–25. 28527533 10.1016/j.jacc.2017.04.052 PMC5491406

[3] Anand S, Bradshaw C, Prabhakaran D. Prevention and management of CVD in LMICs: why do ethnicity, culture, and context matter? BMC Med. 2020;18(1):7. 31973762 10.1186/s12916-019-1480-9 PMC6979081

[4] GBD 2019 Diseases and Injuries Collaborators. Global burden of 369 diseases and injuries in 204 countries and territories, 1990–2019: a systematic analysis for the Global Burden of Disease Study 2019. *Lancet*. 2020;396(10258):1204–22. 10.1016/S0140-6736(20)30925-9 PMC7567026 33069326

[5] Kiatchoosakun S, Sutra S, Thepsuthammarat K. Coronary artery disease in the Thai population: data from health situation analysis 2010. J Med Assoc Thai. 2012;95(Suppl 7):S149-155. 23130447

[6] The Heart Association of Thailand Under the Royal Patronage of H.M. the King. Thai Acute Coronary Syndromes Guidelines 2020 [Internet]. 2020 [cited 2023 Mar 20]. Available from: http://www.thaiheart.org/Thai-ACS-Guidelines-2020.

[7] Keeley EC, Boura JA, Grines CL. Primary angioplasty versus intravenous thrombolytic therapy for acute myocardial infarction: a quantitative review of 23 randomised trials. Lancet. 2003;361(9351):13–20. 12517460 10.1016/S0140-6736(03)12113-7

[8] Chung SC, Gedeborg R, Nicholas O, James S, Jeppsson A, Wolfe C, et al. Acute myocardial infarction: a comparison of short-term survival in national outcome registries in Sweden and the UK. Lancet. 2014;383(9925):1305–12. 24461715 10.1016/S0140-6736(13)62070-X PMC4255068

[9] Khera S, Kolte D, Gupta T, Subramanian KS, Khanna N, Aronow WS, et al. Temporal trends and sex differences in revascularization and outcomes of ST-segment elevation myocardial infarction in younger adults in the United States. J Am Coll Cardiol. 2015;66(18):1961–72. 26515998 10.1016/j.jacc.2015.08.865

[10] Limwattananon C, Jaratpatthararoj J, Thungthong J, Limwattananon P, Kitkhuandee A. Access to reperfusion therapy and mortality outcomes in patients with ST-segment elevation myocardial infarction under universal health coverage in Thailand. BMC Cardiovasc Disord. 2020;20(1):121. 32143572 10.1186/s12872-020-01379-3 PMC7060593

[11] Gajanana D, Weintraub WS, Kolm P, Rogers T, Iantorno M, Ben-Dor I, et al. Trends in death rate 2009 to 2018 following percutaneous coronary intervention stratified by acuteness of presentation. Am J Cardiol. 2019;124(9):1349–56. 31547993 10.1016/j.amjcard.2019.07.059

[12] Kwok CS, Narain A, Pacha HM, Lo TS, Holroyd EW, Alraies MC, et al. Readmissions to hospital after percutaneous coronary intervention: a systematic review and meta-analysis of factors associated with readmissions. Cardiovasc Revasc Med. 2020;21(3):375–91. 31196797 10.1016/j.carrev.2019.05.016

[13] Tangcharoensathien V, Witthayapipopsakul W, Panichkriangkrai W, Patcharanarumol W, Mills A. Health systems development in Thailand: a solid platform for successful implementation of universal health coverage. Lancet. 2018;391(10126):1205–23. 29397200 10.1016/S0140-6736(18)30198-3

[14] Aungkulanon S, Tangcharoensathien V, Shibuya K, Bundhamcharoen K, Chongsuvivatwong V. Post universal health coverage trend and geographical inequalities of mortality in Thailand. Int J Equity Health. 2016;15(1):190. 27876056 10.1186/s12939-016-0479-5 PMC5120448

[15] Longevity TLH. Human rights for healthy longevity. Lancet Healthy Longev. 2023;4(10): e517. 37804837 10.1016/S2666-7568(23)00196-4

[16] Tsao CW, Aday AW, Almarzooq ZI, Alonso A, Beaton AZ, Bittencourt MS, et al. Heart disease and stroke statistics-2022 update: a report from the American Heart Association. Circulation. 2022;145(8):e153-639. 35078371 10.1161/CIR.0000000000001052

[17] Rao C, Porapakkham Y, Pattaraarchachai J, Polprasert W, Swampunyalert N, Lopez AD. Verifying causes of death in Thailand: rationale and methods for empirical investigation. Popul Health Metr. 2010;8:11. 20482758 10.1186/1478-7954-8-11 PMC2880956

[18] Rohrer JM. Thinking clearly about correlations and causation: graphical causal models for observational data. Adv Methods Pract Psychol Sci. 2018;1(1):27–42.

[19] Gu XS, Rosenbaum PR. Comparison of multivariate matching methods: structures, distances, and algorithms. J Comput Graph Stat. 1993;2(4):405–20.

[20] Stuart EA, Lee BK, Leacy FP. Prognostic score-based balance measures can be a useful diagnostic for propensity score methods in comparative effectiveness research. J Clin Epidemiol. 2013;66(8 Suppl):S84-S90.e1. 23849158 10.1016/j.jclinepi.2013.01.013 PMC3713509

[21] Olsson U. Confidence intervals for the mean of a log-normal distribution. J Stat Educ. 2005;13(1):3.

[22] Bradburn MJ, Clark TG, Love SB, Altman DG. Survival analysis Part III: multivariate data analysis—choosing a model and assessing its adequacy and fit. Br J Cancer. 2003;89(4):605–11. 12915864 10.1038/sj.bjc.6601120 PMC2376927

[23] VanderWeele TJ, Ding P. Sensitivity analysis in observational research: Introducing the E-value. Ann Intern Med. 2017;167(4):268–74. 28693043 10.7326/M16-2607

[24] Ho D, Imai K, King G, Stuart E, Whitworth A, Greifer N (2023) MatchIt: nonparametric preprocessing for parametric causal inference [Internet]. [Cited 1 Dec 2023]. Available from: https://cran.r-project.org/web/packages/MatchIt/index.html

[25] Chongsuvivatwong V (2022) epiDisplay: epidemiological data display package [Internet]. [cited 1 Dec 2023]. Available from: https://cran.r-project.org/web/packages/epiDisplay/index.html

[26] Kassambara A, Kosinski M, Biecek P, Fabian S. survminer: Drawing Survival Curves using ‘ggplot2’ [Internet]. 2021 [Cited 1 Dec 2023]. Available from: https://cran.r-project.org/web/packages/survminer/index.html

[27] Wickham H (2023) RStudio. tidyverse: Easily Install and Load the ‘Tidyverse’ [Internet]. [Cited 1 Dec 2023]. Available from: https://cran.r-project.org/web/packages/tidyverse/index.html

[28] Mathur MB, Smith LH, Ding P, VanderWeele TJ (2021) EValue: sensitivity analyses for unmeasured confounding and other biases in observational studies and meta-analyses [Internet]. [Cited 1 Dec 2023]. Available from: https://cran.r-project.org/web/packages/EValue/index.html

[29] Aune D, Schlesinger S, Norat T, Riboli E. Tobacco smoking and the risk of sudden cardiac death: a systematic review and meta-analysis of prospective studies. Eur J Epidemiol. 2018;33(6):509–21. 29417317 10.1007/s10654-017-0351-y PMC5995997

[30] Sendor R, Stürmer T. Core concepts in pharmacoepidemiology: Confounding by indication and the role of active comparators. Pharmacoepidemiol Drug Saf. 2022;31(3):261–9. 35019190 10.1002/pds.5407 PMC9121653

[31] Ki YJ, Han K, Kim HS, Han JK. Smoking and cardiovascular outcomes after percutaneous coronary intervention: a Korean study. Eur Heart J. 2023;44(42):4461–72. 37757448 10.1093/eurheartj/ehad616

[32] Chesebro JH, Knatterud G, Roberts R, Borer J, Cohen LS, Dalen J, et al. Thrombolysis in Myocardial Infarction (TIMI) Trial, Phase I: a comparison between intravenous tissue plasminogen activator and intravenous streptokinase. Clinical findings through hospital discharge. Circulation. 1987;76(1):142–54. 3109764 10.1161/01.cir.76.1.142

[33] Stone GW, Généreux P, Harrington RA, White HD, Gibson CM, Steg PG, et al. Impact of lesion complexity on peri-procedural adverse events and the benefit of potent intravenous platelet adenosine diphosphate receptor inhibition after percutaneous coronary intervention: core laboratory analysis from 10 854 patients from the CHAMPION PHOENIX trial. Eur Heart J. 2018;39(46):4112–21. 30203006 10.1093/eurheartj/ehy562 PMC6284164

[34] Byrne RA, Rossello X, Coughlan JJ, Barbato E, Berry C, Chieffo A, et al. 2023 ESC Guidelines for the management of acute coronary syndromes. Eur Heart J Acute Cardiovasc Care. 2024;13(1):55–161. 37740496 10.1093/ehjacc/zuad107

[35] Fazel R, Joseph TI, Sankardas MA, Pinto DS, Yeh RW, Kumbhani DJ, et al. Comparison of reperfusion strategies for ST-segment–elevation myocardial infarction: a multivariate network meta-analysis. J Am Heart Assoc. 2020;9(12): e015186. 32500800 10.1161/JAHA.119.015186 PMC7429064

[36] Yanamala CM, Bundhun PK, Ahmed A. Comparing mortality between fibrinolysis and primary percutaneous coronary intervention in patients with acute myocardial infarction: a systematic review and meta-analysis of 27 randomized-controlled trials including 11 429 patients. Coron Artery Dis. 2017;28(4):315–25. 28362665 10.1097/MCA.0000000000000489

[37] Zhao YJ, Sun Y, Wang F, Cai YY, Alolga RN, Qi LW, et al. Comprehensive evaluation of time-varied outcomes for invasive and conservative strategies in patients with NSTE-ACS: a meta-analysis of randomized controlled trials. Front Cardiovasc Med. 2023;10:1197451. 37745128 10.3389/fcvm.2023.1197451 PMC10516546

