# Supplementary material for: Impact of access to coronary angiography and percutaneous coronary intervention on in-hospital and five-year mortality in patients with acute coronary syndrome: a propensity-matched cohort study in Thailand
Source: Glob Health Res Policy. 2024 Nov 19;9:48. doi: 10.1186/s41256-024-00390-x (PMC11575078; doi:10.1186/s41256-024-00390-x)
Supplement: Supplementary file 1 — Additional file 1. [file 41256_2024_390_MOESM1_ESM.docx]

**Supplementary Tables**

sTable 1: Underlying diseases/conditions included in matching with their ICD-10 definition

| Disease/condition | ICD-10 diagnoses before the hospitalization due to ACS |
| --- | --- |
| Hypertension | I10 |
| Hypercholesterolemia | E78 |
| Diabetes | E10–14 |
| Hyperthyroidism | C73, E05, P72.1 |
| Hypothyroidism | E0, E23.0, E89.0 |
| Chronic kidney disease | I12–13, N18 |
| Severe obesity | E66 |
| Valvular heart disease | I0, I34–39 |
| Previous TIA/stroke | G45–46, I61–69 |
| Atrial fibrillation | I48 |
| Heart failure | I50 |

ACS, acute coronary syndrome; ICD-10, International Classification of Diseases, Tenth Revision; TIA, transient ischemic attack

sTable 2: Comparison of the characteristics of patients with first STEMI events after excluding in-hospital deaths

| Demographics | Access to  CAG and PCI | No access to CAG and PCI | SMD |
| --- | --- | --- | --- |
| Total, N | 2426 | 1785 |  |
| n (%) |  |  |  |
| Male | 1660 (68.4) | 1245 (69.7) | 0.029 |
| Age, mean (SD) | 65.07 (11.75) | 64.81 (12.86) | 0.021 |
| Hypertension | 1058 (43.6) | 846 (47.4) | 0.076 |
| Hypercholesterolemia | 901 (37.1) | 779 (43.6) | 0.133 |
| Diabetes | 604 (24.9) | 430 (24.1) | 0.019 |
| Hyperthyroidism | 22 (0.9) | 20 (1.1) | 0.021 |
| Hypothyroidism | 32 (1.3) | 30 (1.7) | 0.03 |
| Chronic kidney disease | 307 (12.7) | 271 (15.2) | 0.073 |
| Severe obesity | 5 (0.2) | 5 (0.3) | 0.015 |
| Valvular heart disease | 79 (3.3) | 52 (2.9) | 0.02 |
| Previous TIA/stroke | 110 (4.5) | 79 (4.4) | 0.005 |
| Atrial fibrillation | 159 (6.6) | 103 (5.8) | 0.033 |
| Heart failure | 522 (21.5) | 382 (21.4) | 0.003 |
| Purpose of admission at a PCI-center hospital | | | 0.101 |
| To consider rescue PCI | 2321 (95.7) | 1667 (93.4) |  |
| To consider primary PCI | 105 (4.3) | 118 (6.6) |  |
| Health regional zone |  |  | 0.233 |
| 1 | 277 (11.4) | 203 (11.4) |  |
| 2 | 125 (5.2) | 77 (4.3) |  |
| 3 | 102 (4.2) | 56 (3.1) |  |
| 4 | 100 (4.1) | 66 (3.7) |  |
| 5 | 230 (9.5) | 118 (6.6) |  |
| 6 | 150 (6.2) | 83 (4.6) |  |
| 7 | 99 (4.1) | 56 (3.1) |  |
| 8 | 103 (4.2) | 70 (3.9) |  |
| 9 | 110 (4.5) | 71 (4) |  |
| 10 | 155 (6.4) | 88 (4.9) |  |
| 11 | 372 (15.3)* | 370 (20.7)* |  |
| 12 | 509 (21) | 463 (25.9) |  |
| 13 | 94 (3.9) | 64 (3.6) |  |

CAG, coronary artery angiography; PCI, percutaneous coronary intervention; SD, standard deviation; STEMI, ST-segment elevation myocardial infarction; SMD, standardized mean difference; TIA, transient ischemic attack

sTable 3: Comparison of characteristics of patients with first NSTE-ACS events after excluding in-hospital deaths

| Demographics | Access to  CAG and PCI | No access to CAG and PCI | SMD |
| --- | --- | --- | --- |
| Total, N | 4905 | 4591 |  |
| n (%) |  |  |  |
| Male | 2978 (60.7) | 2746 (59.8) | 0.018 |
| Age, mean (SD) | 64.76 (10.77) | 64.7 (11.98) | 0.005 |
| Hypertension | 3541 (72.2) | 3315 (72.2) | <0.001 |
| Hypercholesterolemia | 3233 (65.9) | 3036 (66.1) | 0.005 |
| Diabetes | 2058 (42) | 1963 (42.8) | 0.016 |
| Hyperthyroidism | 53 (1.1) | 50 (1.1) | 0.001 |
| Hypothyroidism | 106 (2.2) | 105 (2.3) | 0.009 |
| Chronic kidney disease | 908 (18.5) | 899 (19.6) | 0.027 |
| Severe obesity | 35 (0.7) | 23 (0.5) | 0.027 |
| Valvular heart disease | 273 (5.6) | 242 (5.3) | 0.013 |
| Previous TIA/stroke | 166 (3.4) | 145 (3.2) | 0.013 |
| Atrial fibrillation | 364 (7.4) | 360 (7.8) | 0.016 |
| Heart failure | 1400 (28.5) | 1297 (28.3) | 0.006 |
| Type of admission at a PCI-center hospital | | | 0.052 |
| From any department | 4454 (90.8) | 4235 (92.2) |  |
| Referred from other hospital | 451 (9.2) | 356 (7.8) |  |
| Health regional zone |  |  | 0.109 |
| 1 | 364 (7.4) | 374 (8.1) |  |
| 2 | 272 (5.5) | 267 (5.8) |  |
| 3 | 258 (5.3) | 241 (5.2) |  |
| 4 | 548 (11.2) | 513 (11.2) |  |
| 5 | 570 (11.6) | 537 (11.7) |  |
| 6 | 471 (9.6) | 453 (9.9) |  |
| 7 | 309 (6.3) | 296 (6.4) |  |
| 8 | 246 (5) | 273 (5.9) |  |
| 9 | 386 (7.9) | 350 (7.6) |  |
| 10 | 198 (4) | 220 (4.8) |  |
| 11 | 309 (6.3) | 295 (6.4) |  |
| 12 | 269 (5.5) | 258 (5.6) |  |
| 13 | 705 (14.4) | 514 (11.2) |  |

CAG, coronary artery angiography; NSTE-ACS, non-ST-elevation acute coronary syndrome; PCI, percutaneous coronary intervention; SD, standard deviation; SMD, standardized mean difference; TIA, transient ischemic attack

**Supplementary Figures**

sFigure 1: Individuals recruited

CABG, coronary artery bypass grafting; CAG, coronary artery angiography; NSTE-ACS, non-ST-elevation acute coronary syndrome; PCI, percutaneous coronary intervention

*
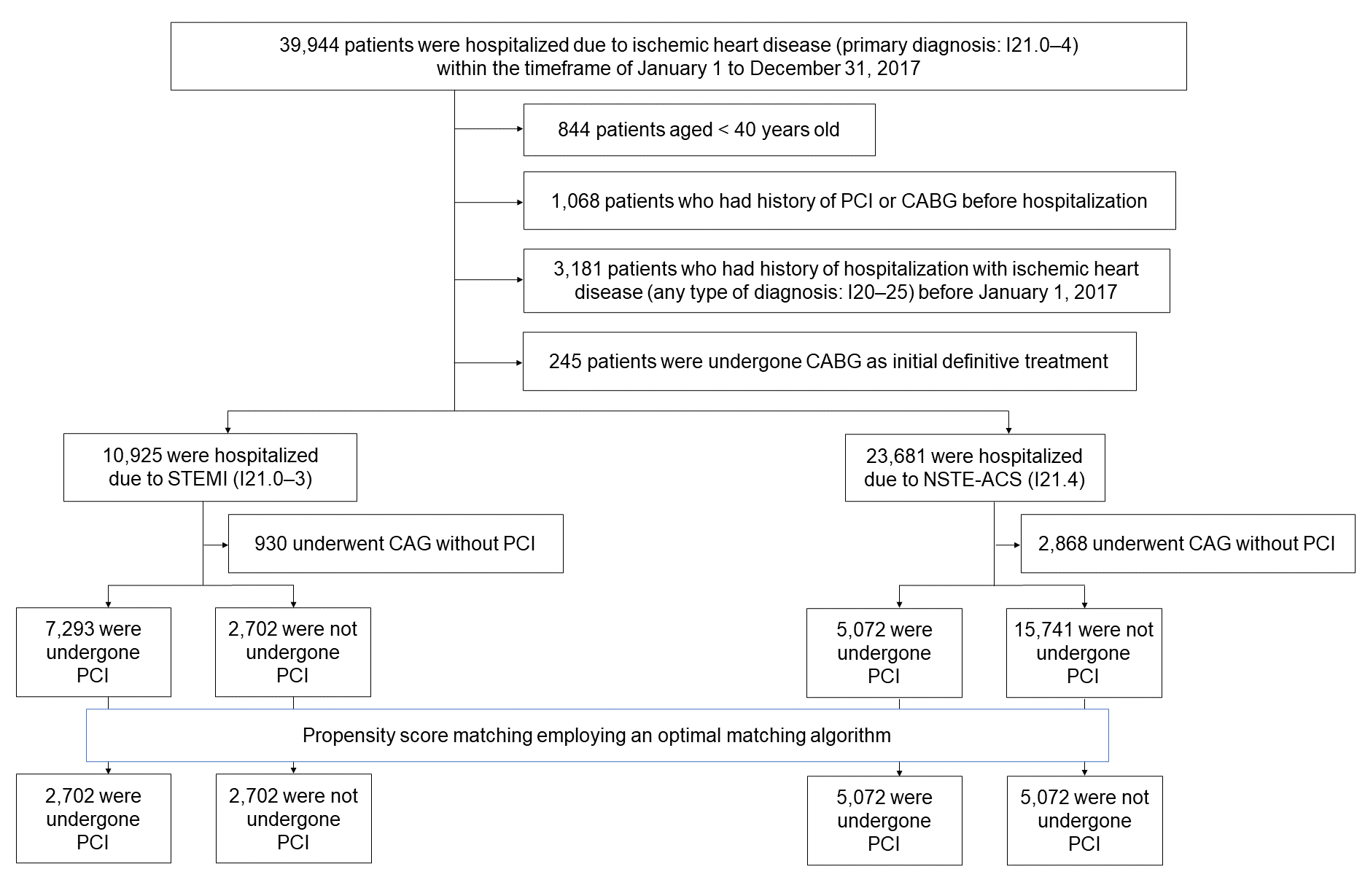
*

sFigure 2: Log-minus-log plot of the hazard function for overall mortality in patients with and without access to CAG and PCI for STEMI


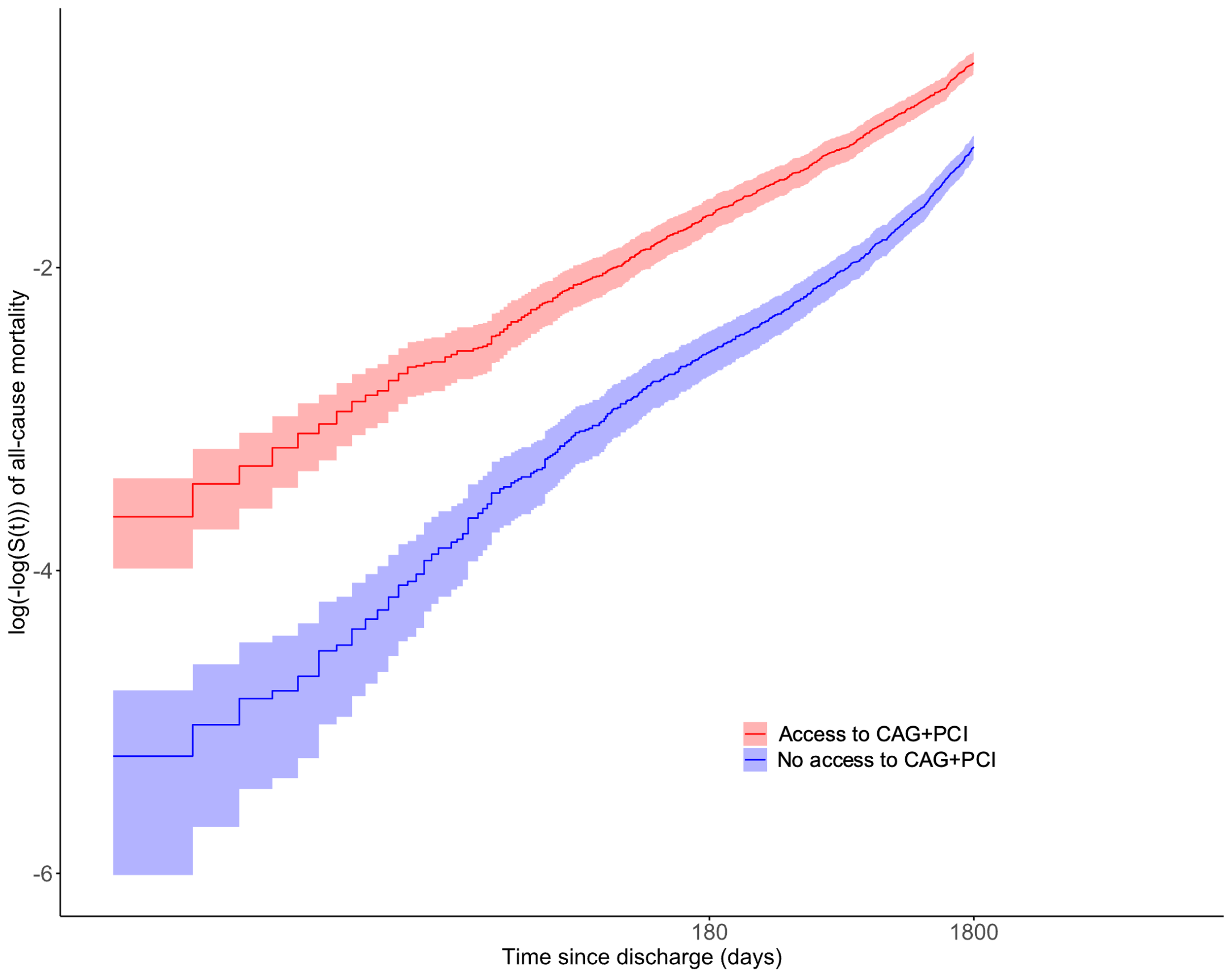


CAG, coronary artery angiography; PCI, percutaneous coronary intervention; STEMI, ST-segment elevation myocardial infarction

sFigure 3: Log-minus-log plot of the hazard function for overall mortality in patients with and without access to CAG and PCI for NSTE-ACS


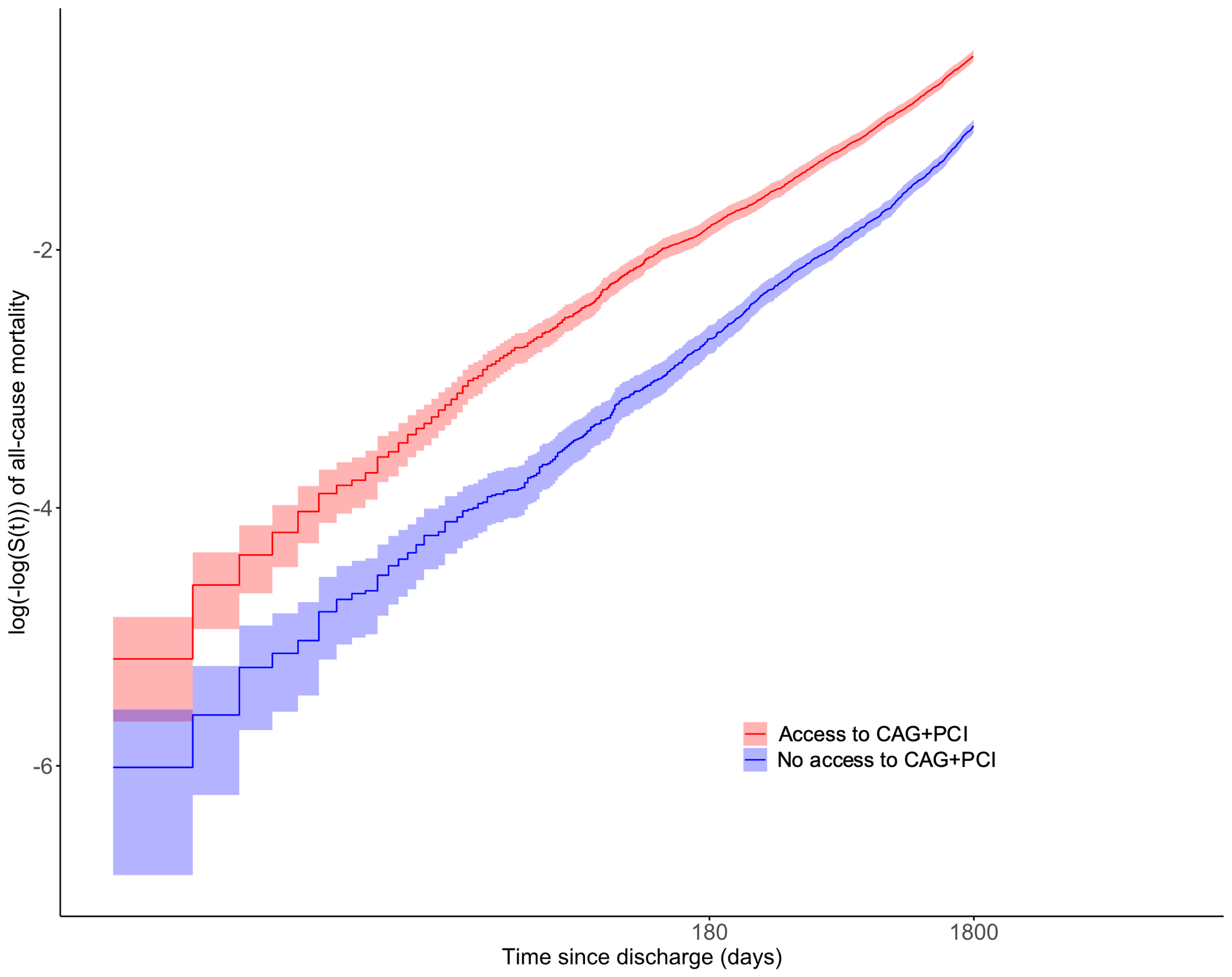


CAG, coronary artery angiography; NSTE-ACS, non-ST-elevation acute coronary syndrome; PCI, percutaneous coronary intervention

sFigure 4: Log-minus-log plot of the hazard function for heart condition-related mortality in patients with and without access to CAG and PCI for STEMI


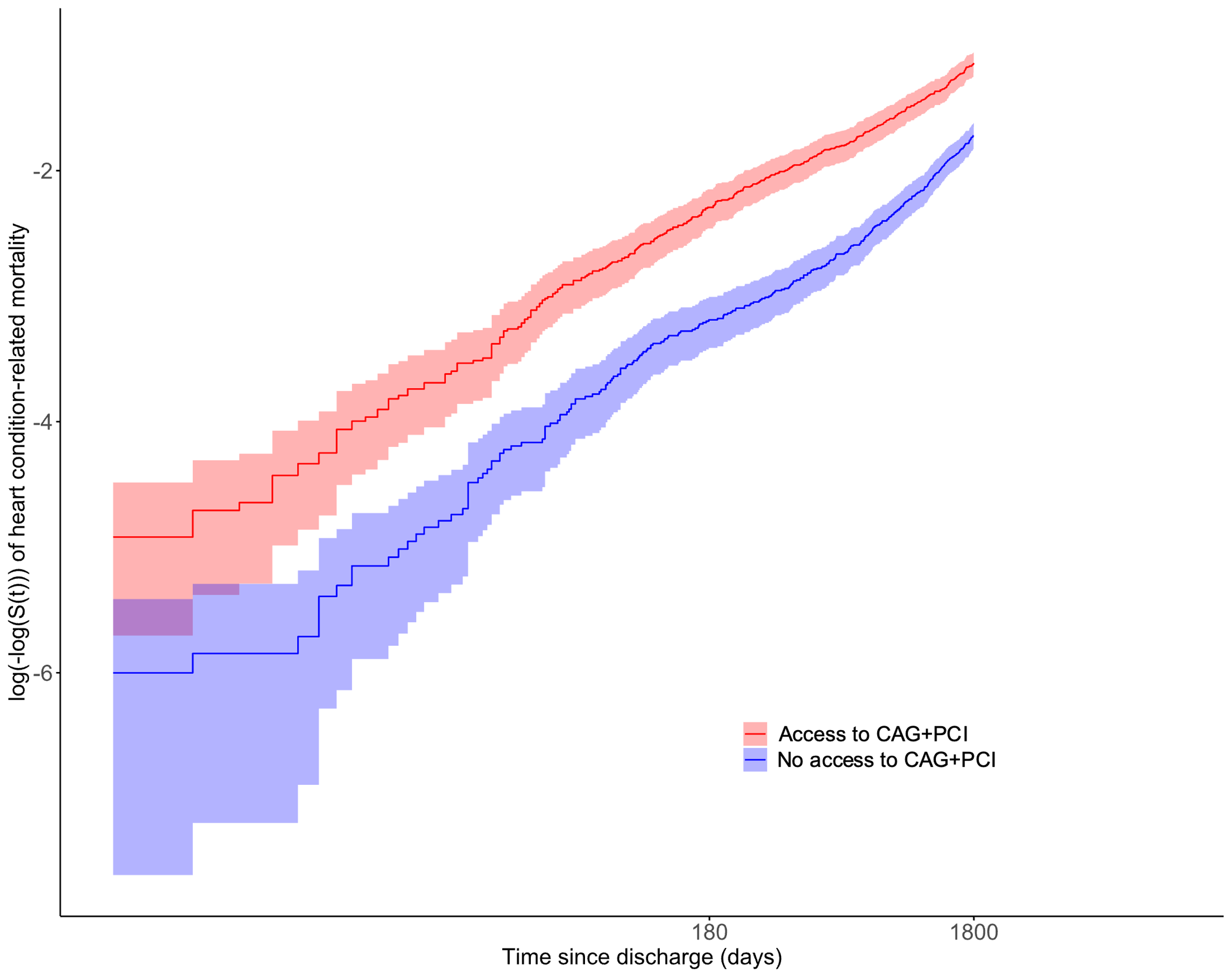


CAG, coronary artery angiography; PCI, percutaneous coronary intervention; STEMI, ST-segment elevation myocardial infarction

sFigure 5: Log-minus-log plot of the hazard function for heart condition-related mortality in patients with and without access to CAG and PCI for NSTE-ACS


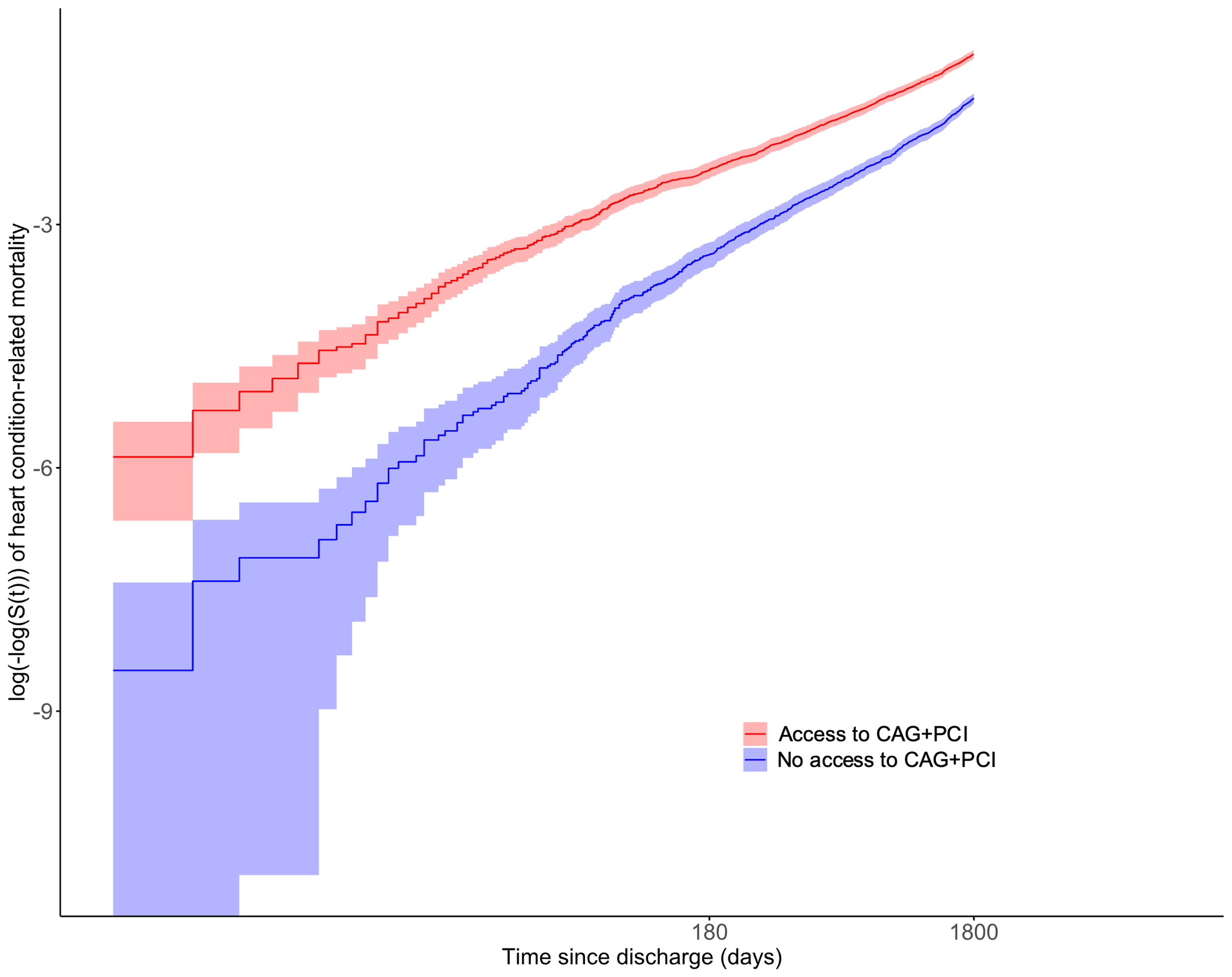


CAG, coronary artery angiography; NSTE-ACS, non-ST-elevation acute coronary syndrome; PCI, percutaneous coronary intervention
